# Piscine Orthoreovirus from Western North America Is Transmissible to Atlantic Salmon and Sockeye Salmon but Fails to Cause Heart and Skeletal Muscle Inflammation

**DOI:** 10.1371/journal.pone.0146229

**Published:** 2016-01-05

**Authors:** Kyle A. Garver, Stewart C. Johnson, Mark P. Polinski, Julia C. Bradshaw, Gary D. Marty, Heindrich N. Snyman, Diane B. Morrison, Jon Richard

**Affiliations:** 1 Pacific Biological Station, Department of Fisheries and Oceans, Nanaimo, British Columbia, Canada; 2 Animal Health Centre, Ministry of Agriculture, Abbotsford, British Columbia, Canada; 3 Marine Harvest Canada, Campbell River, British Columbia, Canada; INRA, FRANCE

## Abstract

Heart and skeletal muscle inflammation (HSMI) is a significant and often fatal disease of cultured Atlantic salmon in Norway. The consistent presence of Piscine orthoreovirus (PRV) in HSMI diseased fish along with the correlation of viral load and antigen with development of lesions has supported the supposition that PRV is the etiologic agent of this condition; yet the absence of an *in vitro* culture system to demonstrate disease causation and the widespread prevalence of this virus in the absence of disease continues to obfuscate the etiological role of PRV with regard to HSMI. In this study, we explore the infectivity and disease causing potential of PRV from western North America—a region now considered endemic for PRV but without manifestation of HSMI—in challenge experiments modeled upon previous reports associating PRV with HSMI. We identified that western North American PRV is highly infective by intraperitoneal injection in Atlantic salmon as well as through cohabitation of both Atlantic and Sockeye salmon. High prevalence of viral RNA in peripheral blood of infected fish persisted for as long as 59 weeks post-challenge. Nevertheless, no microscopic lesions, disease, or mortality could be attributed to the presence of PRV, and only a minor transcriptional induction of the antiviral Mx gene occurred in blood and kidney samples during log-linear replication of viral RNA. Comparative analysis of the S1 segment of PRV identified high similarity between this North American sequence and previous sequences associated with HSMI, suggesting that factors such as viral co-infection, alternate PRV strains, host condition, or specific environmental circumstances may be required to cause this disease.

## Introduction

In 1999, a new and emerging disease syndrome was described by the observation of a unique set of pathological changes in the heart and skeletal muscles of farmed Atlantic salmon *Salmo salar* in Norway [1,2]. This disease, designated heart and skeletal muscle inflammation (HSMI), has become widespread and common among the Atlantic salmon farming industry in Norway and is known to cause increased mortalities as well as high morbidity that can reach upwards of 100% in affected cages [1,3]. Since 2007, over 100 outbreaks have occurred annually in Norwegian Atlantic salmon farms [4].

Through controlled laboratory studies, HSMI has been shown to be infectious. The disease was transmitted to naïve fish by experimental injections with tissue homogenate from HSMI-affected fish and through cohabitation with fish exhibiting HSMI [1,2,5–7]. Electron microscopy of tissues from HSMI diseased fish revealed the presence of virus-like particles suggesting a viral etiology, although *in vitro* virus culture attempts from diseased tissue proved unsuccessful [8]. However, through the use of pyrosequencing, a novel virus denoted piscine orthoreovirus (PRV) was discovered in Atlantic salmon with HSMI [9].

Following the discovery of PRV in fish with HSMI, studies have further suggested a causal relationship between PRV and HSMI by correlating higher viral load and PRV antigen in the heart of fish with HSMI [6,9,10] as well as with the development of heart lesions [11] which has led to the general supposition that PRV is the causative agent of HSMI [7,12,13]. However, whether PRV is truly the etiological agent of HSMI or other disease conditions in farmed Atlantic salmon remains uncertain [9–11,14,15]. An associated relationship to a disease condition does not definitively imply causation, and one of the main limitations with ascertaining a cause and effect relationship with PRV and HSMI stems from a lack of an established *in vitro* culture system for PRV required to fulfill Koch’s postulates. Moreover, the PRV-HSMI connection has been further complicated by that fact that the virus is not only detected in diseased fish but also commonly abundant in apparently healthy individuals without signs of HSMI [11,14]. This is particularly evident outside of Norway where molecular testing has demonstrated PRV to be geographically widespread, with positive detections occurring in Ireland, Chile, United States, and Canada [16,17]. In western North America, PRV detections have occurred not only in farmed Atlantic salmon but also in farmed and wild Pacific salmon and trout species [17–19]. Although the host range of this virus appears primarily restricted to salmonids, there has been the uncommon detection of PRVin a few non-salmonid species such as capelin *Mallotus villosus*, Atlantic horse mackerel *Trachurus trachurus*, and Atlantic herring *Clupea harengus*, and great silver smelt *Argentina silus* [20]. Nevertheless, to our knowledge, HMSI has yet to be confirmed in fish other than from farmed Atlantic salmon in Norway with only one report of a disease resembling HSMI in farmed salmon in Scotland [21].

The occurrence of PRV in a wide range of hosts and geographic locations is particularly noteworthy to consider in the context of evaluating the pathogenicity of PRV. For instance, in Norway, the presence of high loads of PRV in Atlantic salmon has been suggested as a requirement for the development of HSMI because the occurrence of disease has not been reported without the presence of virus. However in western North America, where the presence of high PRV loads is also commonly abundant among wild and farmed salmon, there is no known occurrence of HSMI, suggesting that PRV from western North America is avirulent or that factors other than the presence of PRV are required to cause disease. The context in which a disease occurs is dependent upon the complex interactions of the host, pathogen and their environment. In the case where PRV is not associated with HSMI, as observed in western North America, a unique opportunity is presented to compare and contrast this virus to those circumstances in which PRV has been associated with HSMI.

To this end, we exposed Atlantic and Sockeye salmon *Oncorhynchus nerka* to PRV from western North America under controlled laboratory conditions that reflected previously described studies where PRV was associated with HSMI [1,2,5–7,22]. Exposure studies utilized an intraperitoneal (i.p.) injection route as well as a waterborne challenge through cohabitation with naturally infected Atlantic salmon to systematically evaluate the transmission dynamics and whether challenge with PRV from western North America could result in the development of HSMI or other disease conditions.

## Results

### PRV tissue distribution in naturally infected Atlantic salmon

**Fig 1:**
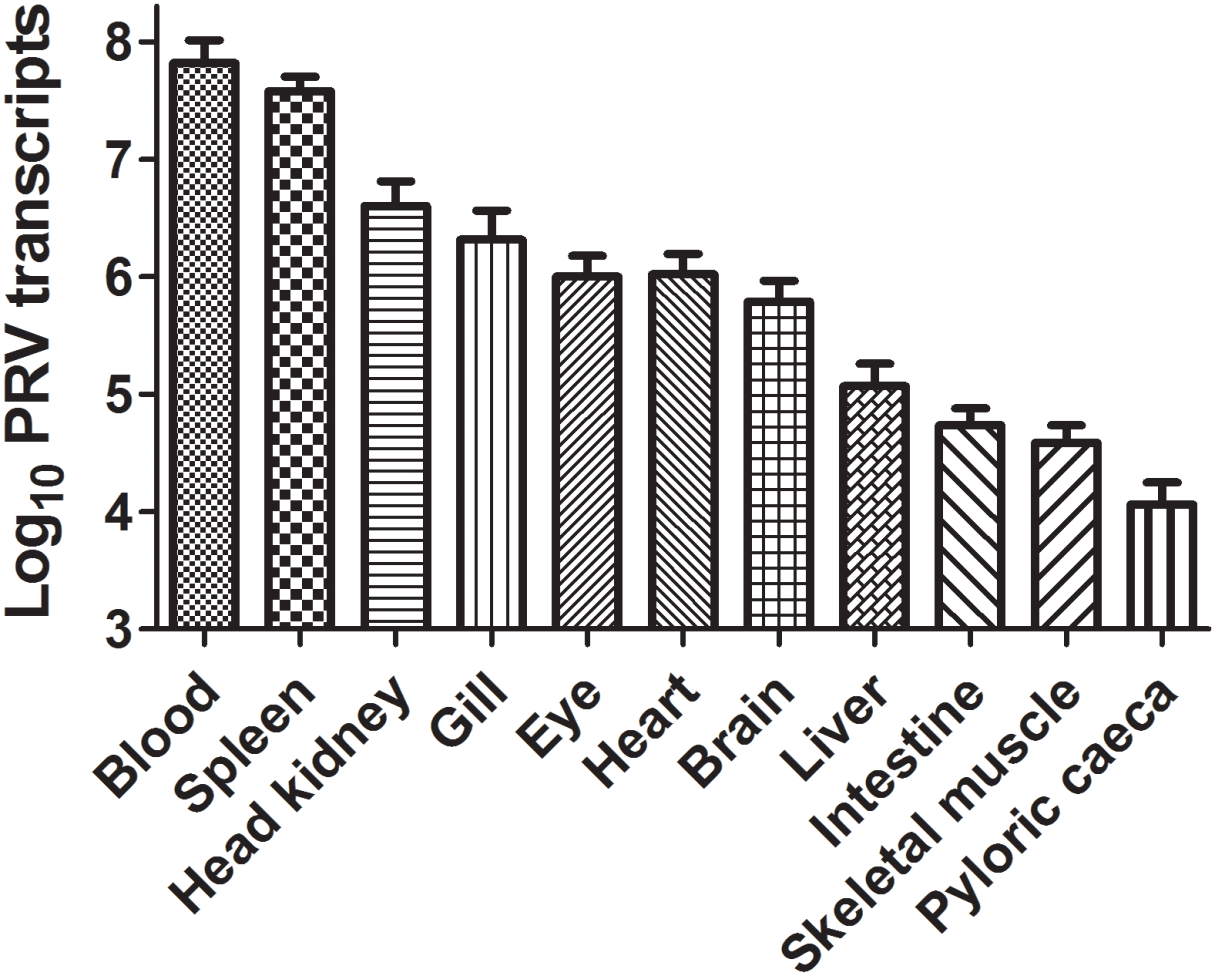
PRV distribution in naturally infected Atlantic salmon. Mean (± SE) copy number of PRV L1 genomic sequence per 1 μg extracted RNA in 11 different Atlantic salmon tissues (N = 7).

The tissue distribution of PRV in North American salmonids without HSMI has not been well documented and thus an initial objective of this study was to evaluate PRV abundance in tissues of naturally infected Atlantic salmon within western North America. In naturally infected Atlantic salmon from a hatchery in British Columbia, Canada, blood, spleen and anterior kidney had the highest relative PRV loads out of 11 tissues tested, corresponding to 6.6 (Ct 11.6), 3.8 (Ct 13.8) and 0.4 × 10^7^ (Ct 19.2) mean copies/μg total RNA, respectively (Fig 1).

### Response of Atlantic salmon to PRV infection by i.p. injection

**Fig 2:**
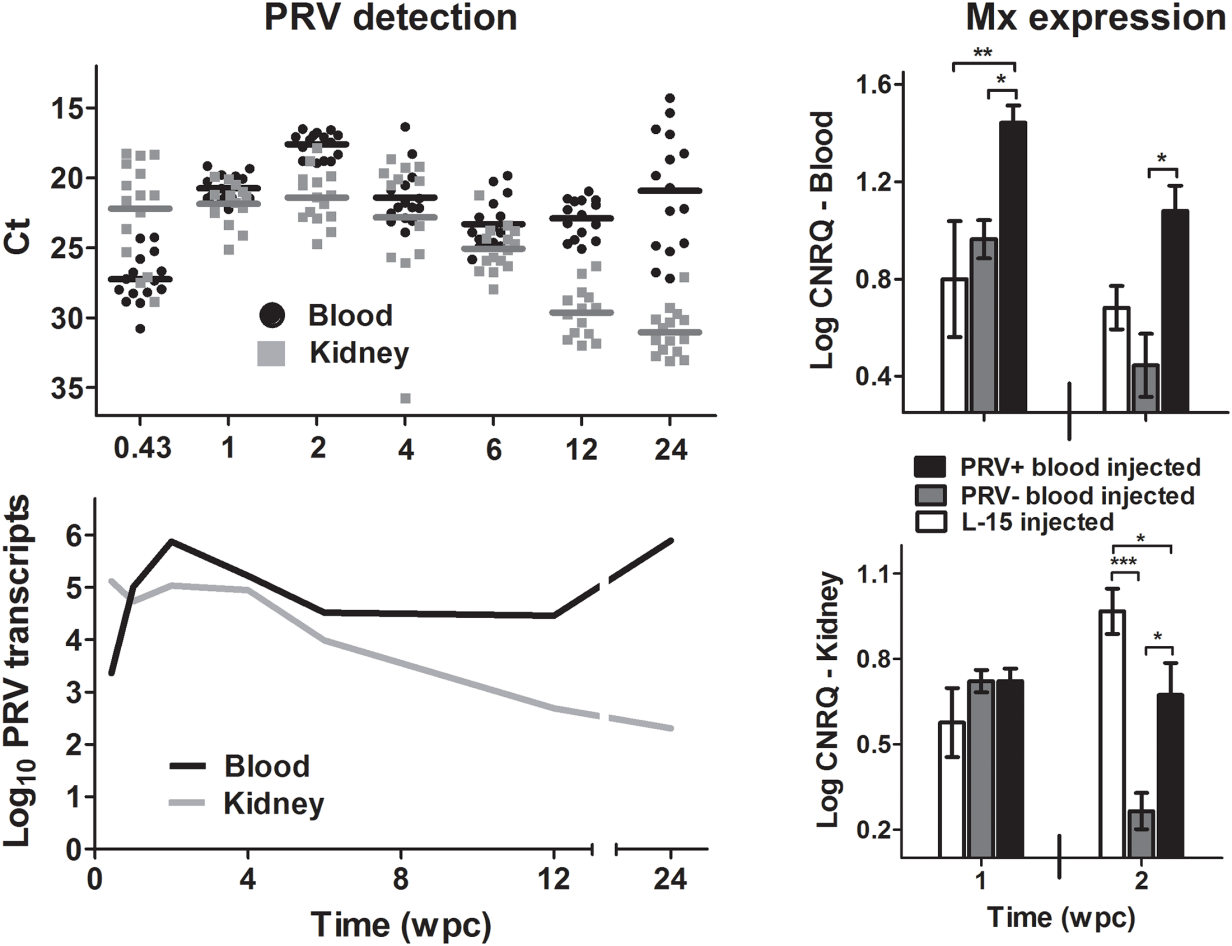
PRV load and antiviral-related gene expression in Atlantic salmon following injection challenge. The relative individual threshold cycles (Ct) and treatment mean (horizontal bar) obtained by qPCR analysis for all blood and anterior kidney samples obtained during 24 weeks post challenge (wpc). Mean copy number of PRV L1 genomic sequence per 1 μg extracted RNA is also provided in time course, as well as the corrected normalized relative quantity (CNRQ) of Mx antiviral mRNA transcripts for each of three treatment groups at either 1 or 2 wpc for both peripheral blood and anterior kidney tissues. For gene expression analyses, significant (* p<0.05; ** p<0.01; *** p<0.001) differences in expression between treatment groups are indicated at each time point.

Inoculums prepared from PRV+ Atlantic salmon experiencing HSMI in Norway have induced HSMI in naïve Atlantic salmon via intraperitoneal injection [6,7] and induced host antiviral transcriptional responses [6]. In this study, we attempted to emulate these conditions using PRV+ inoculums prepared from naturally infected Atlantic salmon in British Columbia. Following injection, blood and kidney tissues of naïve Atlantic salmon became heavily infected with PRV, and log-linear replication of viral specific L1 transcripts was detected in peripheral blood between 3 and 14 days post injection (Fig 2). Fourteen days after injection, fish were transitioned to saltwater; thereafter, viral loads subsided until a resurgence of viral RNA occurred in a portion of blood samples at 24 weeks post challenge (wpc). Interestingly, at one and two wpc, when viral loads in both blood and anterior kidney were highest, gene expression analysis of Mx mRNA transcripts—a gene involved in the classic antiviral interferon response pathway and previously shown to be up-regulated in response to HSMI [6]–showed only modest up-regulation in infected blood samples relative to naïve blood-injected or media-injected controls (Fig 2). Mx expression was slightly less pronounced in anterior kidney, showing that although Mx transcription was significantly greater in infected samples relative to naïve blood-injected controls at two wpc, both treatments were significantly lower than media-injected controls and all were less than a two-fold change relative to the median value across treatments; thereby bringing into question the biological significance for PRV associated Mx transcription within this tissue. Further, during this challenge the fish did not develop changes in hematocrit, HSMI, increased mortality, or other signs of disease attributable to PRV infection (details of histopathological findings are in supporting information (S1 File); details of the entire sample inventory including viral load, hematocrit, and mortality results are in supporting information (S2 File)). Most of the microscopic lesions in these fish are relatively common among growing young Atlantic salmon and sockeye salmon reared under intensive culture conditions. Three of the fish had severe, acute, hepatocellular hydropic degeneration: one had been injected with PRV- negative inoculum and two had been injected with PRV+ inoculum (S1 File). These lesions are not common in cultured fish, and they probably are a result of suboptimal fish handling or environmental conditions in the 24 h before sampling.

### Transmission potential of PRV by cohabitation

**Fig 3:**
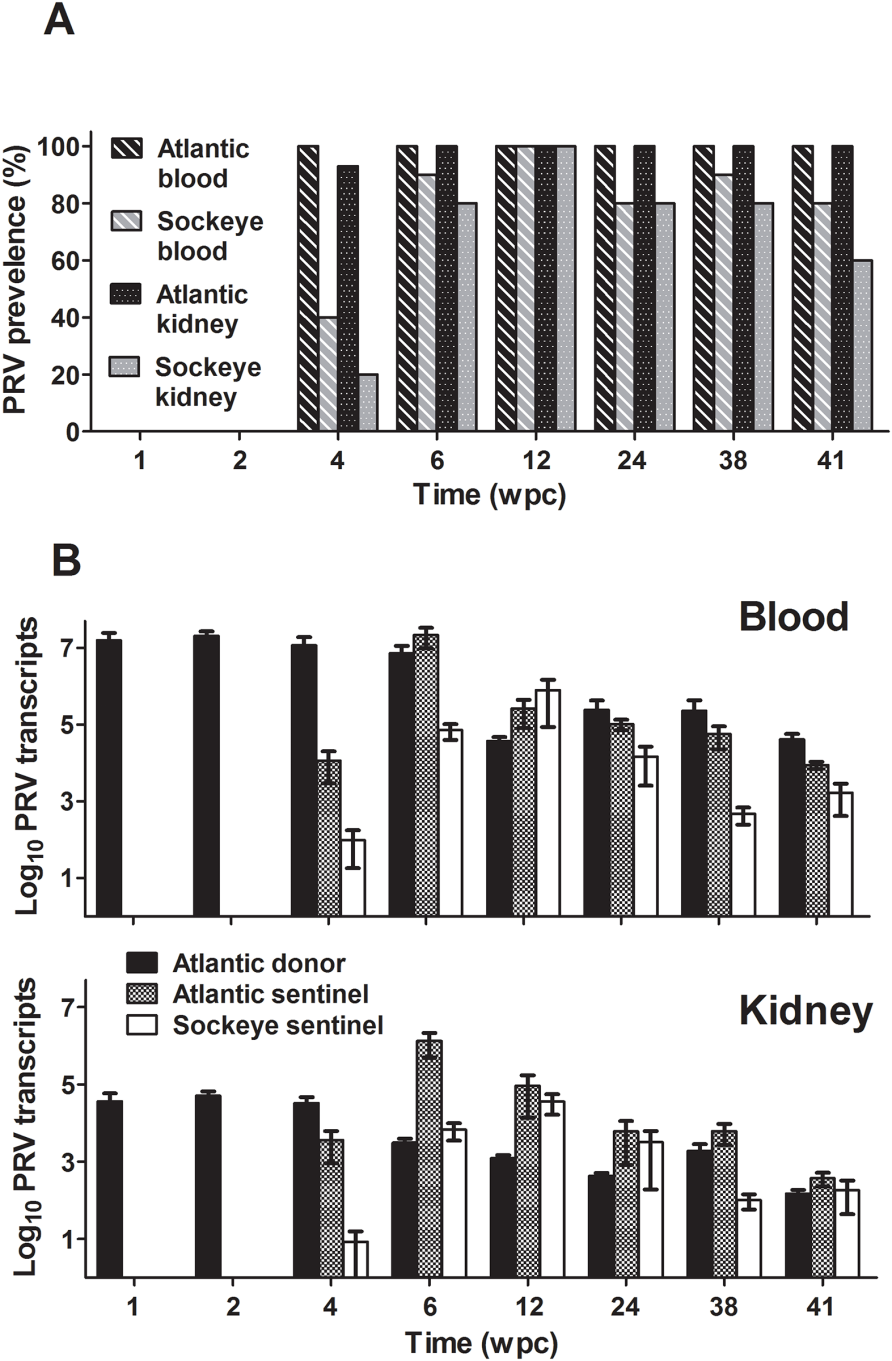
PRV prevalence and relative load in cohabitated Atlantic and Sockeye salmon. Proportion of blood and anterior kidney samples infected with PRV (prevalence) within sampled subpopulations (Atlantic salmon N = 15; Sockeye salmon N = 10) for each treatment group over a period of 41 weeks (A). The mean copy number of PRV L1 genomic sequence per 1 μg extracted RNA (±SE) is also provided for peripheral blood and anterior kidney (B).

In Norwegian research laboratories, PRV and HSMI have been transmitted through cohabitation [5, 7], consequently with the detection of PRV in western North America concern was raised as to whether the virus and any associated disease can spread from infected populations to naïve wild and domestic stocks. To investigate PRV transmission potential, naturally infected PRV+ Atlantic salmon (donors) were cohabitated in sea water with PRV- Atlantic and Sockeye salmon (sentinels). Atlantic salmon sentinels became infected with PRV following four weeks of cohabitation and remained infected throughout the 41-week challenge period (Fig 3). High viral loads in both the blood and anterior kidney of sentinel fish were comparable to if not higher than the loads reached by i.p. injection. Sockeye salmon tended to be slightly less susceptible to PRV than Atlantic salmon; viral load did not become as high in either kidney or blood samples compared to Atlantic salmon, infections appeared to take longer to develop, and some individuals appeared refractory or were able to clear PRV infections. Nevertheless, most Sockeye became infected by eight weeks of cohabitation and infections persisted throughout the 41 week study period. Similar to i.p. injection challenge of Atlantic salmon, no increased hematocrit, HSMI, increased mortality, or other signs of disease could be attributed to PRV infection in any Atlantic or Sockeye salmon during this trial (S1 and S2 Files). Among the nine fish that died during the experiment and were subjected to necropsy, four had no obvious cause of death and five fish had significant microscopic lesions, but the suite of lesions in each fish was different. None of these nine fish had inflammation in the heart or skeletal muscle (S1 File). Some of the significant lesions in these fish also occur in fish with infectious diseases; however, the sections contained no obvious infectious agents, and the low prevalence and lack of consistent lesions are evidence against a common infectious cause.

### Infectious potential of persistent PRV

**Fig 4:**
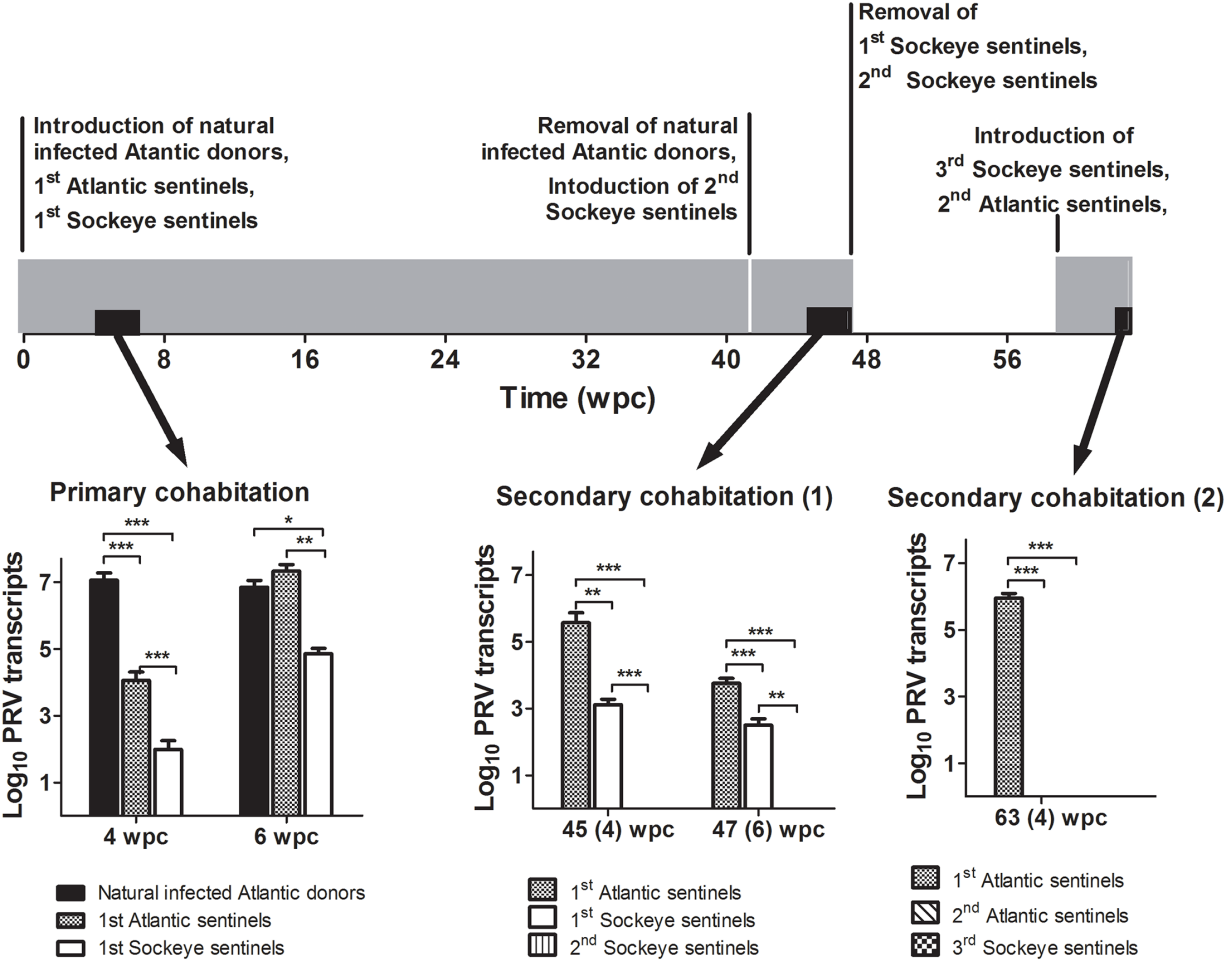
Infectious potential of PRV in cohabitated Atlantic and Sockeye salmon. A time line outlines three successive cohabitation experiments (Gray bars) as well as the introduction and removal of treatment groups. The relative PRV L1 transcriptional load (±SE) is also provided at three intervals (black bars) either 4 or 6 weeks from the start of each of the three successive challenges. Significant (* p<0.05; ** p<0.01; *** p<0.001) differences in viral load are indicated at each time point.

PRV specific RNA persisted for long periods in Atlantic and Sockeye salmon in both our i.p. injection (24 weeks) and cohabitation (41 weeks) trials. To determine the infectious potential of this RNA, PRV+ Atlantic and Sockeye salmon sentinels following the initial 41 week cohabitation experiment were separately re-cohabitated with newly introduced naïve Sockeye salmon sentinels. Further, a portion of PRV+ Atlantic salmon sentinels from the initial cohabitation experiment were held an additional 18 weeks (59 weeks post PRV exposure) before naïve Atlantic and Sockeye salmon were introduced for cohabitation. In both instances, PRV specific L1 transcripts persisted in infected individuals; however, viral transcripts could not be detected in newly introduced sentinels (Fig 4). As with previous trials, no HSMI or associated lesions were detected in PRV infected fish (S1 File). To ensure age-matched sentinels introduced at these later time points were still susceptible to PRV infection, inoculum prepared from PRV positive blood collected from naturally infected donors six weeks after the start of the first cohabitation experiment was injected into a portion of age-matched naïve fish. Injection challenged fish experienced increased viral loads in both anterior kidney and blood samples (S2 File).

### Comparison of PRV S1 genome segments

**Fig 5:**
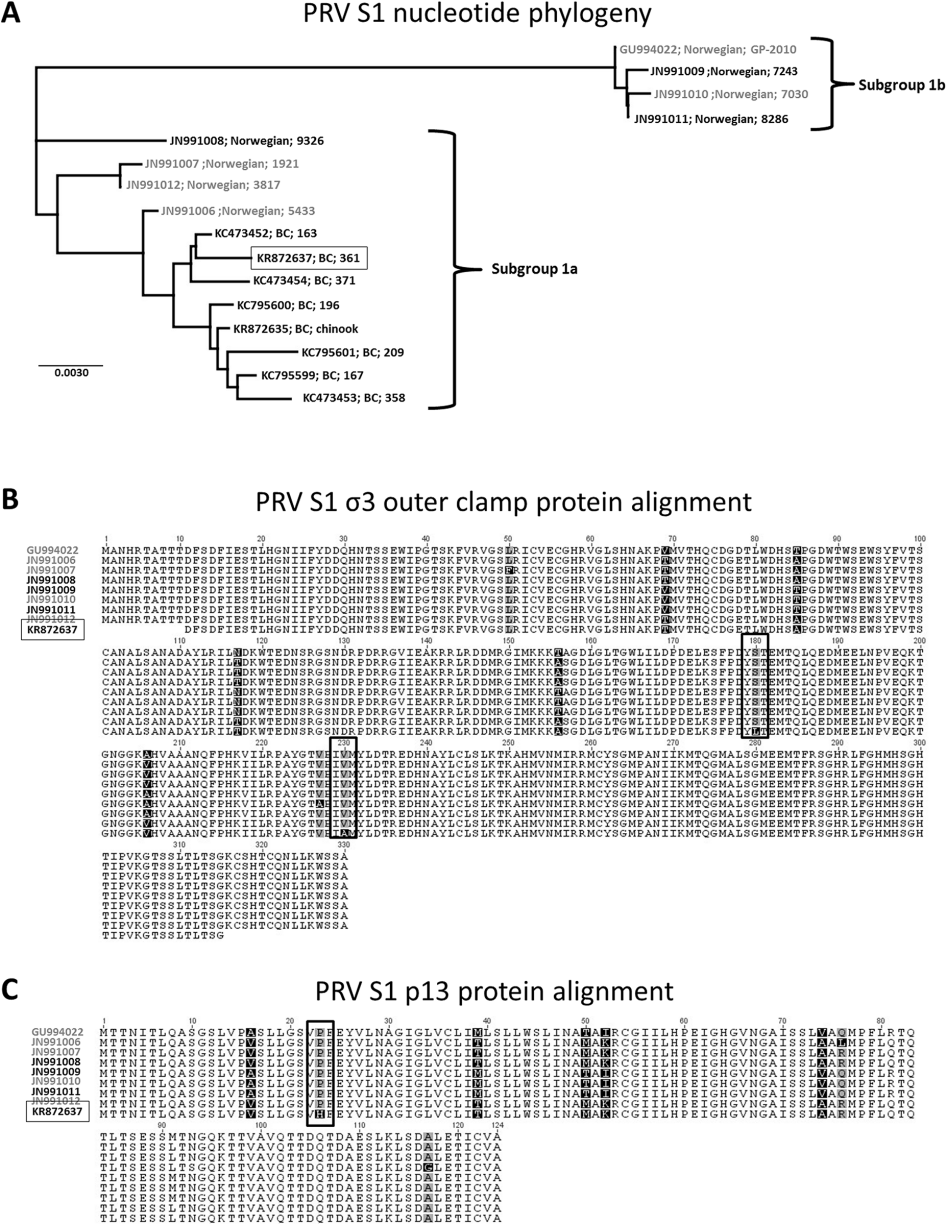
PRV S1 segment phylogenetic and protein comparisons between HSMI and non-HSMI associated isolates. Phylogenetic Jukes-cantor neighbor-joining comparison of previously published sequences from Norway and Canada with the Canadian sequence (boxed) of PRV used in this study (A). Predicted amino acid alignments of both the σ3 (B) and p13 (C) proteins identify unique substitutions (vertical boxes) for the Canadian sequence of PRV in this study compared to eight Norwegian PRV sequences. In all cases, sequences of PRV obtained from HSMI disease fish (gray) are distinguished from non-HSMI associated variants (black).

Previous phylogenetic comparisons led to the hypothesis that western North American sequences of PRV have recently diverged from European sequences [17]. As genetic variation could present a possible explanation for the variability in virulence between the North American and Norwegian PRV to cause HSMI, we compared nucleotide and predicted protein translation of the PRV S1 segment used in this study to other previously published sequences from both Norway and British Columbia with contrasting association with HSMI [9,11,17,18]. The S1 segment is a region considered to be both high in strain-specific diversity [17] and associated with virulence in reovirus infection [21]. Phylogenetic comparisons revealed that the partial S1 sequence identified from the PRV used in this study grouped with other BC PRV sequences within the Ia subgroup (Fig 5A) and had 99.5% sequence identity to the most closely related Norwegian PRV associated with HSMI. Predicted protein translation comparison of the current BC PRV to eight previously sequenced Norwegian PRV identified two amino acid substitutions in the σ3 and one amino acid substation in the p13 translation unique to the BC isolate; however, these substitutions where the same in comparison to either isolates from HSMI or non-HSMI diseased fish and thus could not be used to predict disease state (Fig 5B&5C). Further, predicted protein structure comparisons of the current BC PRV and HSMI associated sequence types demonstrated minimal structural variation which in many cases was less pronounced than differences observed between PRV sequences associated with HSMI (S1 Fig).

## Discussion

In this study, western North American PRV was shown to be highly infectious in Atlantic and Sockeye salmon. PRV was easily transmitted to these species whereby persistent infections were established with up to 100% prevalence. Virus infection was followed by periods of significant viral amplification and shedding which demonstrated the capacity of North American PRV to complete its life cycle in either Atlantic or Pacific salmonids. However, a complete lack of associated lesions and mortality in infected populations exhibiting high viral loads also indicates that western North American PRV is non-pathogenic. These finding are further supported by previous reports purporting the avirulent nature of PRV in western North America where high viral loads in the absence of disease was observed [18,19]. Nevertheless, apart from lacking the occurrence of disease, our observations show that western North American PRV behaves strikingly similar to other PRV isolates that have been associated with HSMI with regard to infectivity. For instance, HSMI experimentally induced in Atlantic salmon by cohabitation with HSMI diseased fish identified PRV RNA at high levels in the peripheral blood [7] similar to observations in our study; yet as in our study, changes in hematocrit have not been observed due to the presence of replicating virus [1,7]. Further as observed in our studies the transmission of western North American PRV to naïve cohabitants within weeks after exposure, is analogous to the temporal occurrence of PRV infections in cohabitation studies leading to HSMI in Atlantic salmon [6,7]. With fundamental infectious processes of western North American PRV resembling those of PRV found associated with HSMI, it is curious as to what factor(s) could account for such pathogenic differences. Is the lack of disease observed in our studies herein simply a reflection of not employing an adequate infectious dose? Were the host populations employed in our study adequately susceptible? Is the absence of HSMI herein a result of viral strain differences between PRV in western North America verses those associated with HSMI in Norway or is the absence of HSMI herein evidence that PRV is not the sole causative agent?

For other aquatic viral pathogens, such as infectious hematopoietic necrosis virus (IHNV), infectious outcomes are often highly dose dependent. For instance, lethal disease in Atlantic salmon caused by IHNV requires waterborne virus concentrations in excess of 10 plaque forming units per ml [23]. The minimum infectious dose of PRV required for the formation of HSMI is unknown; however, Finstad *et al*. [7] demonstrated that a inoculum generated from tissues of HSMI diseased Atlantic salmon with a mean PRV Ct of 25.6 was sufficient to induce signs of HSMI within 10 wpc. In our injection challenge of Atlantic salmon, a PRV+ inoculum at a Ct of 19.6 was used. Presuming equivalent detection efficiencies based on similar quantitative assessment methods, this represents a 64-fold higher PRV dose than one shown to induce HSMI in Norway. Although RNA based quantitation methods do not give indication of infectious virions, comparable inoculum preparation techniques and similar temporal increases in viral RNA loads following challenge in these two studies suggests that the lack of HSMI development in our challenge was not likely due to an insufficient exposure dose of infectious PRV.

Epidemiology of HSMI outbreaks in Norwegian farmed Atlantic salmon has identified that fish are commonly affected 5 to 9 months after transfer to sea, evidence that host life stage or saltwater exposure time might be important factors for disease [1]. Risk mapping of HSMI has also revealed farmed Atlantic salmon to be critically susceptible to exposure at small sizes [24]. Consequently, we initiated PRV infections in Atlantic salmon pre-smolts during this study and followed viral kinetics and disease progression throughout the physiologically demanding stage of smoltification to capture this critical life stage and reflect the common temporal nature of HSMI in Norway. In this study, initial viral loads were comparable to those previously reported in PRV cohabitation challenge of Atlantic salmon which developed HSMI (peak mean Atlantic salmon blood Ct ~18.5 at 8 wpc [7] versus peak mean Atlantic salmon blood Ct 17.1 at 6 wpc in this study), identifying that populations of Atlantic salmon used in this study were sufficiently susceptible and could sustain PRV infections at a level previously associated with HSMI. It is, therefore, unlikely that host susceptibility to PRV was a contributing factor towards the lack of HSMI development during these current investigations. Nonetheless, we cannot rule out genetic differences between British Columbian and Norwegian stocks of Atlantic salmon as a potential factor influencing development of HSMI. In BC, one of three recognized industry strains, is of Norwegian origin (Mowi) however since importation the Mowi strain has under gone significant mixing with other strains originating from Scotland and Quebec. Furthermore, geographic variation and some genetic drift has likely attributed to differences between the Atlantic salmon strains of British Columbia and Norway [25]. Future work exposing Norwegian origin Atlantic salmon to western N American PRV will be required to fully understand the impact of host genetics on development of HSMI

Another important consideration must be given to isolate-specific genetic differences which could impact virulence. In some instances, viral associated disease can be attributable to a single genetic component which can be strain specific, as exemplified by the HPR0 variant of infectious salmon anemia virus (ISAV) where a single deletion in the highly polymorphic region (HPR) of the virus genome changes the virus from a highly virulent disease agent to a ubiquitous non-pathogenic variant [26,27]. In the case of PRV, molecular epidemiology investigations comparing PRV sequences generated from HSMI diseased fish verses non diseased fish demonstrated highly similar sequences without consistent amino acid or nucleotide variations to facilitate identification of a unique virulent PRV strain responsible for HSMI development [11,17]. Likewise, in a comparison of the western North American PRV S1 sequence utilized in our study with previous S1 sequences of PRV associated with HSMI a high degree of similarity was revealed between our isolate and those associated with HSMI. It is possible that further detailed analysis may reveal genetic determinants of virulence in other regions of the genome and thus continued genomic comparisons and assessment between PRV isolates is needed. However, in cases where virulent and avirulent strains exist in other aquatic fish viruses the genetic variants are usually accompanied with distinct tropism and replication kinetics, as further exemplified by localized gill infections of HPR0-ISAV relative to the systemic and severe nature of classical ISAV disease [28]. As noted from our exposure studies herein, the capacity of western North American PRV to infect erythrocytes and reach high viral loads as observed with Norwegian PRV suggests that the avirulent nature of western North American PRV is not a consequence of the strain’s inability to infect and replicate in its host. Nevertheless, further research aimed at directly comparing western North American PRV with PRV that has been associated with HSMI is required to fully assess if there are molecular indicators of virulence associated with this virus.

A new and somewhat unexpected finding in the current study was the apparent loss of peer-to-peer transmission that developed during persistent PRV infections. Neither Atlantic nor Sockeye salmon that were infected with PRV by cohabitation for a period of greater than 37 weeks could transmit infection to naïve Sockeye or Atlantic salmon during six weeks of further cohabitation. This loss in infectivity may be a consequence of reduced viral load. During primary infectious cohabitation shedding fish had a mean viral blood load of 7.15 × 10^6^ L1 copies/μg RNA at 6 wpc, whereas the mean load at 6 weeks in shedding fish during two subsequent secondary cohabitation challenges was <0.01 and 0.89 × 10^6^ L1 copies/μg RNA, respectively (Fig 4). This suggests that highly infected individuals may be required to initiate peer-to-peer transmission of this virus. In previous reports concerning PRV and HSMI, abundance of PRV L1 transcripts persisted at higher levels in farmed fish with HSMI than in cohorts free of disease [11]. Cohabitation of HSMI infected fish also resulted in peak PRV transcription to coincide with the highest host Mx transcriptional response and heart lesions in naïve sentinels at approximately 9–10 wpc [6]. In the present study where HSMI was not observed, PRV genomic transcripts reached their highest load in Atlantic salmon blood and kidney tissues between four and eight weeks post cohabitation and two weeks post injection followed by a slow trend of decline until resurgence in some blood samples at the end of each challenge (Figs 2 and 3). Additionally, Mx transcription during a period of log-linear increases in viral RNA in peripheral blood showed only modest up regulation (<4 fold) in this study (Fig 2). This is in contrast with transcriptional changes observed previously in heart tissues experiencing myocarditis indicative of HSMI (30–100 fold, [6]). It is hard to make direct transcriptional comparisons between discrete cell populations; however, as blood cells appear to be a primary target for PRV replication [7] and infiltrating blood-associated cells are likely responsible for increases in Mx transcription relative to cardiac myocytes during myocarditis based on viral tropisms, it is curious as to why PRV would cause such an elevated antiviral transcriptional response in heart tissues. Based on the culmination of current data, it could alternatively be speculated that the Mx antiviral response initiated during the inflammatory processes of HSMI may be mostly independent of PRV. Instead, the transcriptional and pathological responses during HSMI could be caused by a discrete agent (such as a co-infecting virus) that produces a conducive state for continued opportunistic replication of PRV that may or may not contribute to disease.

In conclusion, we demonstrate through both injection and cohabitation exposure that western North American PRV is capable of infecting and replicating to high quantities in blood and kidney of Atlantic and Sockeye salmon without causing disease. Infection dynamics and the genetic makeup of PRV from this study were highly similar to previous reports of Norwegian PRV in association with HSMI, and other factors beyond the presence of PRV might be required to initiate a disease state in salmonid hosts. These findings also support a growing body of evidence suggesting that at least in western North America, PRV may simply be a ubiquitous virus found in opportunistic scenarios in diseased fish [18,29]. Caution is therefore needed for current interpretations of the influence of PRV during manifestations of disease such as HSMI, as important components regarding disease causation are as yet unknown, and virulent manifestations of PRV in concert with cofactors such as viral co-infection [30], host condition, or specific environmental circumstances will need careful future consideration in understanding the role of PRV in diseases such as HSMI.

## Materials and Methods

### Ethics Statement

All work with animals was performed in strict accordance with the recommendations in the Canadian Council on Animal Care (CCAC) Guide to the Care and Use of Experimental Animals. The protocols were approved by the Pacific Region Animal Care Committee (Animal Use Protocol Number: 13–013 and 15–003). All fish handling was performed under Aquacalm^™^ (Syndel Laboratories Ltd) or tricaine methanesulfonate (MS222) anesthesia.

### Fish Sources and Husbandry

PRV+ and PRV- populations of Atlantic salmon were sourced from separate commercial freshwater salmon hatcheries on Vancouver Island, British Columbia and brought to the Pacific Biologic Station (PBS) in Nanaimo, British Columbia, Canada. The hatchery supplying PRV–Atlantic salmon had no known historical detection of PRV and pre-transport screening of 20 fish via RT-qPCR proved negative for PRV. Fish were maintained in UV treated partial seawater (10° ± 1°C, salinity 7 ppt) for 5 months prior to their transition to undiluted UV treated seawater (10° ± 1°C, 32 ppt). The PRV+ Atlantic salmon were obtained as smolts and pre-transport screening via RT-qPCR of 47 fish demonstrated 100% positive for PRV. Upon arrival at PBS, fish were placed immediately into seawater (10° ± 1°C, 32 ppt) upon arrival at PBS. Both PRV+ and PRV–populations were held under a natural photoperiod and fed dry pellets (EWOS) at 1% body weight per day prior to challenge. PRV–fish used in the i.p. injection challenge provided the tissue source for the negative inoculum control during that challenge, and were used as sentinels for evaluating transmission of PRV through cohabitation. PRV+ fish provided the tissue source for the PRV+ inoculum in the injection challenge, were used as PRV donor fish during the cohabitation challenge, and were used for preliminary PRV tissue tropism investigations.

Sockeye salmon fry (Sakinaw Lake stock; PRV–) were obtained from Rosewall Creek Hatchery, Fanny Bay, British Columbia and transferred to PBS. Fish were reared in 6°C (± 1°C) dechlorinated freshwater under a natural photoperiod for one year prior to transitioning to seawater, where they were held for 12–26 weeks prior to challenge. These Sockeye salmon were utilized as sentinels during the cohabitation and persistent PRV infection potential challenges described below. Fish were initially fed daily at 2.8% body weight and subsequently transitioned to 1% at time of smolting.

Additional stocks of PRV–(sentinel) Atlantic salmon and Sockeye salmon were obtained near the completion of the cohabitation study for use as newly smolted sentinels in the final persistent PRV infectious potential cohabitation experiment. The Atlantic salmon, transported from a commercial hatchery, were held at PBS in UV treated partial seawater (10°C, 6 ppt) for 13 days and subsequently at a salinity of 14.3 ppt for one month prior to their transition to undiluted UV treated seawater (32 ppt) at the start the first persistent infection challenge. The PRV–Sockeye salmon (Pitt River stock) were obtained from Inch Creek Hatchery and reared at PBS in 6°C (± 1°C) dechlorinated freshwater under a natural photoperiod for ten months prior to introduction to undiluted seawater.

### PRV detection and quantification

Real-time RT-qPCR was performed using previously designed forward and reverse primers and TaqMan probe targeting the L1 fragment of the PRV genome ([9], Table 1). RNA was extracted from all samples using TRIzol Reagent (Life Technologies, ThermoFisher, CA, USA) and eluted in 50 μL RNAse/DNase-free water (Life Technologies) as per manufacturer’s instructions. Eluted RNA was denatured for 1 min at 80°C and 1.5 μg was reverse-transcribed using a High Capacity cDNA Reverse Transcription kit (Life Technologies) following manufacturer’s instructions. Undiluted cDNA was then used as template for qPCR analysis in a StepOne-Plus real-time detection system (Applied Biosystems). Each reaction contained 400 nM primers (Eurofins Genomics) and 300 nM TaqMan probe (Life Technologies), 1X TaqMan Universal Master Mix and 2.5μL template in a total of 25 μL. Cycling conditions included an initial incubation of 94°C for 15 minutes followed by 45 cycles of 94°C for 15s, 54°C for 30s and 72°C for 15s. Samples were assayed in duplicate and were considered positive if both technical replicates reported a Ct value < 40 cycles. Absolute PRV quantification was determined in each instance by serial dilution of a 482 bp double-stranded DNA gBLOCK fragment (IDT Technologies) consisting of sequence targeted by the qPCR primer and probe (S2 Fig). A seven-step 10-fold dilution series of the gBLOCK fragment spanning a dynamic range of 10−10^7^ target copies per reaction was incorporated in duplicate into each run. The limit for accurate quantitative assessment using this technique was calculated to be approximately 50 copies with a limit of detection of ≤5 copies per reaction as previously described [31].

**Table 1:** Oligonucleotide primers and probes used in PRV experiments.

Target	Reference	Forward primer (5’→ 3’)	Reverse primer (5’→ 3’)	Probe (5’→ 3’)
PRV L1	[9]	TGCTAACACTCCAGGAGTCATTG	TGAATCCGCTGCAGATGAGTA	FAM-CGCCGGTAGCTCT-MGBNFQ
PRV S1	[18]	GATAAAGACTTCTGTACGTGAAAC	GATGAATAAGACCTCCTTCC	
Mx	[12]	GATGCTGCACCTCAAGTCCTATTA	CACCAGGTAGCGGATCACCAT	
Β-actin	[5]	TTGCGGTATCCACGAGAC	TAGAGGGAGCCAGAGAGG	
EF1-α	[32]	TGATTGTGCTGTGCTTAC	AACGCTTCTGGCTGTAGG	
RPL2	[5]	TAACGCCTGCCTCTTCACGTTGA	ATGAGGGACCTTGTAGCCAGCA	

### PRV Sequencing

A segment of the S1 region of PRV was targeted for genomic sequencing from seven naturally infected Atlantic salmon utilized in this study using methods previously described [18]. The resulting 1017 bp product was purified using ExoSAP-IT PCR clean-up kit (Affymetrix, CA, USA) and submitted to ACGT Corp. for sequencing (ACGT Corp.,Toronto, Ontario). Amino acid sequence was deduced from resulting nucleotide composition using Geneious R6 molecular analysis software and both nucleotide and amino acid similarities were compared to previously published PRV sequences using the Basic Local Alignment Search Tool (BLAST) on Genbank using Geneious alignment software.

### PRV tissue screening during natural infection

Samples of gill, skeletal muscle, eye, heart, blood, spleen, liver, kidney, pyloric caeca, brain and intestine were individually collected and immediately frozen in liquid nitrogen from seven Atlantic salmon assumed to be infected with PRV. Tissues were stored at -80°C for one week and subsequently homogenized in TRIzol extraction buffer (Life Technologies) using 5 mm steel beads and TissueLyser II (Qiagen) for 2 min at 25 Hz. RNA was extracted from the resulting homogenate as described by the TRIzol manufacturer. PRV detection and quantification was conducted using RT-qPCR as described above.

### PRV infection of Atlantic salmon by i.p. injection

#### Inoculum preparation

Anterior kidney and heart tissues from 21 highly infected Atlantic salmon were selected based on having the lowest Ct scoring (highest viral load) out of a pre-screen of 40 fish. The selected PRV+ tissues were pooled and diluted 1:5 (w:v) in Leibovitz L-15 cell culture media supplemented with 50 μg mL^-1^ gentamycin and homogenized using a Polytron (Kinematica). Homogenate was centrifuged at 2500 × *g* for 10 min at 4°C with the resulting supernatant filtered through a 0.45 μm low protein binding filter (Millipore) and stored on ice until injections. PRV- control inoculum was prepared from kidney and heart tissues of 20 fish confirmed PRV free by RT qPCR. The mean load of PRV L1 transcripts in the PRV+ inoculum was 3.5 × 10^6^ copies/250 μL injection (Ct 19.6).

#### Intraperitoneal injection and monitoring

Atlantic salmon (~ 75 g each) were anesthetized in an aqueous solution of Tricaine methanesulfonate (0.05 g/L) and given a 250 μl intraperitoneal injection of either PRV+ inoculum, PRV–inoculum, or L-15 (120 fish per treatment). Fish were placed in triplicate tanks and held in 11°C (± 1°C) freshwater for 16 days where after the freshwater supply was replaced with sand filtered UV-treated 11°C seawater (32ppt) for the remainder of the 24 week study. Clinical signs of disease, mortality, water temperature and salinity were recorded daily.

#### PRV associated sampling

Blood and tissue samples were collected from five fish per tank (fifteen fish per treatment group) following 3 days and 1, 2, 4, 6, 12, and 24 wpc. Blood samples (~0.5mL) were collected using a 22 ga needle and 3mL syringe and assessed for PRV by qPCR diagnostic screening at all sampled time points as described above. Hematocrit was measured at 24 wpc. For all sampled time points, brain, gill, anterior kidney, heart, liver, and spleen were aseptically collected into RNAse-free tubes and immediately frozen in liquid nitrogen and stored at –80°C prior to RNA extraction. For histopathology, a sample of eye, brain, gill, spleen, heart, liver, anterior kidney, pyloric caeca, intestine, and muscle/skin were collected at 6, 12 and 24 wpc. These tissues were fixed in 10% neutral buffered formalin for 24 h and then transferred to 70% ethanol until further processing. Paraffin blocks were prepared and sections of 3 μm were stained with haemotoxylin and eosin for light microscopy as previously described [19].

#### Mx transcriptional expression

A portion (15μg) of RNA extracted from 36 samples of head kidney and blood (representing six replicates for each i.p. challenged inoculum group at 1 and 2 wpc) that was not reverse transcribed for the detection of PRV was purified using 2 U of DNase I (Life technologies) at 37°C for 45 min followed by RNeasy MinElute Cleanup (Qiagen) as per manufacturer’s instructions. RNA quality was visualized on a 1% bleach denaturing gel and 1.5 μg of each sample was reverse transcribed using a High Capacity cDNA Reverse Transcription Kit (Life Technologies) without RNase inhibitor where the Random Primer mix was substituted with 50μM Olido d(T)_16_. All real-time qPCR analyses were conducted on a StepOnePlus real-time detection system using SYBR green chemistry. Each PCR reaction consisted of 2X SYBR mastermix (Life Technologies), forward and reverse primers (500 nM each; Table 1), and 2 μL cDNA template to a final volume of 20 μL. Samples were assayed in duplicate with a five-step, fourfold dilution series of pooled cDNA included in each run to calculate amplification efficiency and linearity. Cycling conditions consisted of an initial activation of DNA polymerase at 95°C for 10 min, followed by 40 cycles of 5 s at 95°C, 25 s at 60°C, and 10 s at 72°C. At the end of the cycling protocol melt curve analysis was run to ensure amplification specificity. Mx expression was normalized to three reference genes using qbase+ analysis software [33] and the corrected normalized relative quantity was compared at each time point by a two-way ANOVA and Tukey post hoc test using Graphpad Prism 5.0 following Log transformation of the data.

### Transmission of PRV through cohabitation

PRV+ Atlantic salmon donors (N = 171), with adipose fins removed for identification, were divided into three 2000 L cylindrical tanks containing 11°C sand filtered and UV-treated seawater. In one tank, 115 PRV–Atlantic salmon sentinels were added. Each of the other two tanks received 60 Atlantic salmon and 67 Sockeye salmon PRV–sentinels. Each tank was monitored daily and tissue and blood samples were collected from 5 fish per treatment group in each tank at 1, 2, 4, 6, 12, 24, 26, 39 and 41 wpc as for the i.p. injection challenged described above. Hematocrit was measured at 24, 38 and 41 wpc. Sockeye salmon sentinels were sampled at 2, 4, 6, 12 and 41 wpc for histopathology, while donor and sentinel groups of Atlantic salmon were sampled for histopathology at 12 and 41 wpc.

### Infectious potential of persistent PRV

Immediately following the 41 week cohabitation experiment described above, groups of 5 Atlantic and Sockeye salmon sentinels from the previous cohabitation experiment which had become infected with PRV were transferred in duplicate to tanks containing 10 PRV- age matched naïve Sockeye salmon sentinals (2^nd^ group Sockeye sentinels; Fig 4). After 4 weeks of cohabitation, non-lethal blood samples were obtained from all fish, following anesthesia with TMS (0.05 g/L). At 6 weeks post cohabitation, all fish were euthanized with a lethal dose of TMS (0.2 g/L) and samples of blood and head kidney were extracted, frozen in liquid nitrogen and stored at –80°C until analyzed for viral load. Additionally, 40 Atlantic salmon sentinels which had become infected with PRV during the first cohabitation trial were held for an additional 18 weeks (59 weeks post PRV cohabitation exposure) and moved to a new tank containing 20 naive Atlantic and Sockeye salmon sentinels (2^nd^ group Atlantic sentinels, 3^rd^ group Sockeye sentinels; Fig 4). At 6 weeks post cohabitation, all fish were euthanized and samples of blood and head kidney were extracted, frozen in liquid nitrogen and stored at -80 until analyzed for viral load. Susceptibility of age-matched sentinel was confirmed by intraperitoneal injection of 15 sentinel Atlantic salmon and 14 sentinel Sockeye salmon with 100μl of diluted whole blood supernatant (1:4 w:v in L-15 media) from naturally infected PRV+ Atlantic salmon sampled at 4 to 6 weeks following the commencement of the first cohabitation challenge. For analyses of PRV infection, a subset (n = 5) of each of the injected salmon species were euthanized and sampled for blood and head kidney at day 13 post challenge with the remainder of the fish sampled at day 42 post challenge.

## Supporting Information

S1 Fig Deduced amino acid and predicted secondary structure of proteins ơ3 and p13 generated from open reading frame consensus sequences of the bicistronic S1 gene of Piscine orthoreovirus. Amino acid sequences and secondary structures predicted using Geneious R6 molecular analysis software. Alpha helix, beta strand, coil and turn are presented in purple cylinders, yellow arrows, grey sinusoids and blue curved arrow.
(TIF)

S2 Fig Sequence of the 482 bp gBlock fragment used for absolute PRV quantification by RT-qPCR analysis. PRV L1 sequences targeted by the PRV qPCR primer and probe are included in the gBlock yielding an 81 bp amplicon.
(PDF)

S1 File Data file detailing histopathological findings in Atlantic and Sockeye salmon exposed to Piscine orthoreovirus via injection or cohabitation challenge. (XLSX)

S2 File Complete sample inventory detailing the fish number, treatment group, sampling date and associated metrics for each fish collected from designated experiments. (XLSX)
