# Supplementary figures and images for: Piscine Orthoreovirus from Western North America Is Transmissible to Atlantic Salmon and Sockeye Salmon but Fails to Cause Heart and Skeletal Muscle Inflammation
Source: PLoS One. 2016 Jan 5;11(1):e0146229. doi: 10.1371/journal.pone.0146229 (PMC4701501; doi:10.1371/journal.pone.0146229)

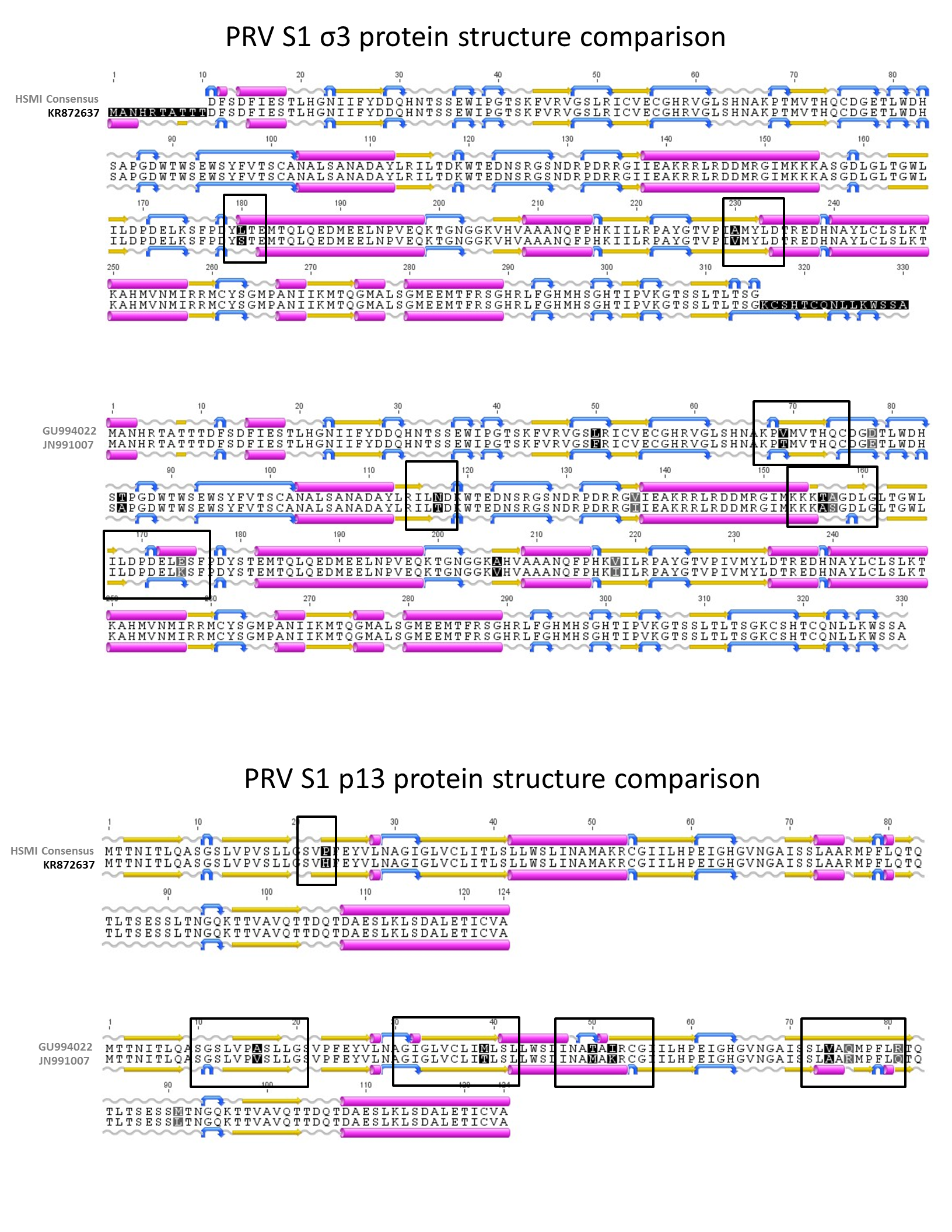

Supplement: S1 Fig — Amino acid sequences and secondary structures predicted using Geneious R6 molecular analysis software. Alpha helix, beta strand, coil and turn are presented in purple cylinders, yellow arrows, grey sinusoids and blue curved arrow. (TIF) [file pone.0146229.s001.tif]
