# Supplementary material for: Piscine Orthoreovirus from Western North America Is Transmissible to Atlantic Salmon and Sockeye Salmon but Fails to Cause Heart and Skeletal Muscle Inflammation
Source: PLoS One. 2016 Jan 5;11(1):e0146229. doi: 10.1371/journal.pone.0146229 (PMC4701501; doi:10.1371/journal.pone.0146229)
Supplement: S2 Fig — PRV L1 sequences targeted by the PRV qPCR primer and probe are included in the gBlock yielding an 81 bp amplicon. (PDF) [file pone.0146229.s002.pdf]

S2 Fig. PRV L1 gBLOCK fragment used in qPCR analysis.

Annotated sequence:

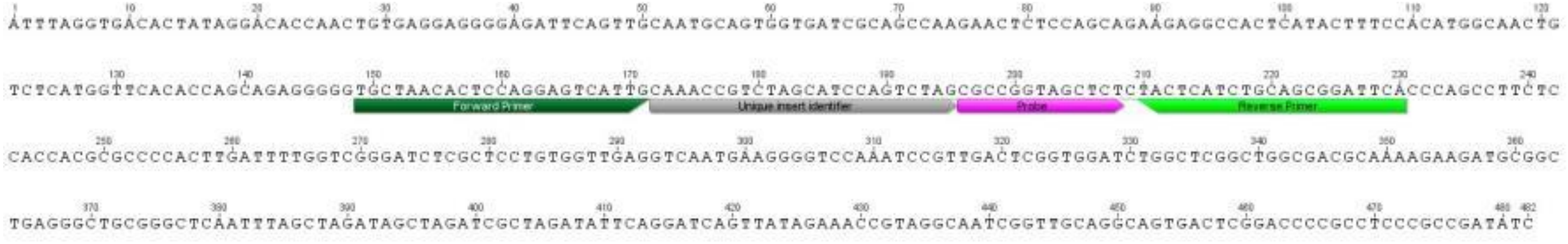

Raw sequence:

```
ATTTAGGTGACACTATAGGACACCAACTGTGAGGAGGGGAGATTCAGTTGCAATGCAGTGGTGATCGCAGCCAAGAACTCTCCAGCAGAAGAGGCCACTCATACTTT
CCACATGGCAACTGTCTCATGGTTCACACCAGCAGAGGGGGTGCTAACACTCCAGGAGTCATTGCAAACCGTCTAGCATCCAGTCTAGCGCCGGTAGCTCTCTACTC
ATCTGCAGCGGATTCACCCAGCCTTCTCCACCACGCGCCCCACTTGATTTTGGTCGGGATCTCGCTCCTGTGGTTGAGGTCAATGAAGGGGTCCAAATCCGTTGACT
CGGTGGATCTGGCTCGGCTGGCGACGCAAAAGAAGATGCGGCTGAGGGCTGCGGGCTCAATTTAGCTAGATAGCTAGATCGCTAGATATTCAGGATCAGTTATAGAA
ACCGTAGGCAATCGGTTGCAGGCAGTGACTCGGACCCCGCCTCCCGCCGATATC
```
